# Attack of the bots: Lessons from a compromised online MSM survey

**DOI:** 10.1017/S095026882510040X

**Published:** 2025-08-15

**Authors:** Abson Madola, Michael DeWitt, Jennifer Wenner, Candice Joy McNeil

**Affiliations:** 1 Section on Infectious Diseases, Wake Forest University School of Medicine, Winston-Salem, NC, USA; 2 Department of Biology, Wake Forest University, Winston-Salem, NC, USA

**Keywords:** survey, data integrity, internet research, MSM, HIV, public health, LGBTQ+, health disparities, data quality

## Abstract

Anonymous online surveys using financial incentives are an essential tool for understanding sexual networks and risk factors including attitudes, sexual behaviors, and practices. However, these surveys are vulnerable to bots attempting to exploit the incentive. We deployed an in-person, limited audience survey via QR code at select locations in North Carolina to assess geolocation application use among men who have sex with men to characterize the role of app usage on infection risk and behavior. The survey was unexpectedly posted on a social media platform and went viral. Descriptive statistics were performed on repeat responses, free-text length, and demographic consistency. Between August 2022 and March 2023, we received 4,709 responses. Only 13 responses were recorded over a 6-month period until a sharp spike occurred: over 500 responses were recorded in a single hour and over 2,000 in a single day. Although free-text responses were often remarkably sophisticated, many multiple-choice responses were internally inconsistent. To protect data quality, all online surveys must incorporate defensive techniques such as response time validation, logic checks, and IP screening. With the rise of large language models, bot attacks with sophisticated responses to open-ended questions pose a growing threat to the integrity of research studies.

Online surveys serve as an effective tool for studying populations with diverse risk profiles and health outcomes, including gay, lesbian, bisexual, queer, plus (GLBQ+) populations. Internet-based surveys are a valuable tool for engaging individuals from diverse backgrounds and lived experiences to better understand differences in health risks and outcomes shaped by social and structural factors [1]. Online surveys offer rapid deployment, efficient data entry, and a more scalable approach to data collection than face-to-face methods, enabling broader participation and more representative sampling across diverse populations [2]. With rapid advancements in artificial intelligence, such as ChatGPT and other large language models, infiltration of these internet-based surveys by autonomous “bots” has increasingly threatened data integrity [3]. Bots are malicious software applications that can be programmed to autonomously and fraudulently submit numerous survey responses, often in an attempt to exploit financial incentives [4]. If not detected quickly, bots can overwhelm researchers with thousands of fraudulent responses in a matter of hours – making financial compensation nearly impossible [5].

Bot interference in online research introduces an additional structural barrier that limits the ability to engage with communities that have been historically excluded from research, thereby reinforcing inequities in health outcomes [6]. This paper describes our experience with bot infiltration in an online survey designed to explore the experiences of men who have sex with men (MSM) on geosocial networking apps (i.e. Grindr, Scruff, etc.). Drawing from this experience, we propose strategies to mitigate bot interference and preserve data integrity, ensuring internet-based epidemiologic research remains a reliable method for studying populations with different risks and outcomes.

We designed an anonymous REDCap (Research Electronic Data Capture) survey to better understand the experiences of MSM on geosocial networking apps, hereafter referred to as apps, and how their experiences influence their human immunodeficiency virus (HIV) risk, mental health, and self-esteem. This Wake Forest University School of Medicine IRB-approved survey was initially advertised via flyers with a QR code and link to the survey displayed at select clinical locations in a large southeastern tertiary hospital and community locations in the Piedmont Triad area of North Carolina (community health clinics, public health department). The flyer advertised a $10 gift card for completion of the survey. The survey was intended to be a small pilot study with a maximum of 100 participants. To be included in the study, participants were required to be at least 18 years old, identify as MSM, and able to read and write in English. The 37-item survey began with demographic questions such as “How old are you?” and “What city do you live in?” with free textboxes followed by multiple choice questions for participants to define their demographics and sexual orientation. The body of the survey asked specific multiple choice and short answer questions that explored sexual history, app usage (frequency of usage, characteristics of partners from the apps, etc.) followed by open ended questions that encouraged participants to write about their experiences in the open text box (e.g. Do you feel that your race/ethnicity impacts your interactions on the app(s)? If so, please explain.). A summary of survey question themes is provided in Table 1. At the end of the anonymous survey, participants were offered the opportunity to complete a separate, unlinked survey to enter their contact information to receive a $10 gift card.

**Table 1.** Survey question themes

Section	Themes covered
Demographics	Age, location, race/ethnicity, gender identity, education level, sexual orientation, outness, HIV testing history, PrEP awareness, HIV status awareness
Sexual history	Recent sexual activity, types of sex practiced, gender identity of partners, race/ethnicity of partners, frequency of barrier protection use
GSN app usage & experiences	Use of geosocial networking apps, app(s) used, partner sourcing through apps, HIV status of partners, motivations for app use, time spent on apps, emotional and psychological impact, perceived racial/ethnic bias, and open-ended reflections

When the survey was unexpectedly posted on a social media platform and quickly went viral, the survey was closed and taken offline. We used time series analysis to track the number of respondents over time. Descriptive statistics were performed on the frequency of repeated responses, length of free text responses, and agreement among demographic and baseline variables. All survey responses were individually reviewed by a member of the study team.

A total of 4,709 responses were captured between 11 August 2022 (date of first response) and 2 March 2023 (date survey was taken offline). Only 13 responses were recorded over a six-month period, until 24 February 2023, when the survey link was posted on social media and a clear change occurred: over 500 responses were recorded in a single hour and over 2,000 in a single day (Figure 1a). The time between responses rapidly decreased from weeks to minutes after the survey went viral (Figure 1b). The initial responses were primarily from within 30 miles of the recruitment site and the remaining responses included many out-of-state and international locales as well as non-geographic answers. The first 13 responses appeared to be from genuine human participants - evidenced by consistent, logical and thoughtful answers. For example, in response to the question, “Please describe how your time on the app(s) makes you feel about yourself. a participant responded, “I stopped using the apps recently and I felt like I have more control about how I perceive myself.” In contrast, multiple choice and open text responses from likely bots were often illogical and contained many internally inconsistent responses. In some cases, identical answers to free response survey questions were seen across multiple respondents. For example, in response to the same question, “Please describe how your time on the app(s) makes you feel about yourself,” 24 participants responded, “I feel happy, I can do the real myself.” All 24 of these responses were recorded within the same minute. Additional features suggestive of bot activity included use of non-English characters, responses irrelevant to the question, and respondents listing a city outside of North Carolina.

**Figure 1.**
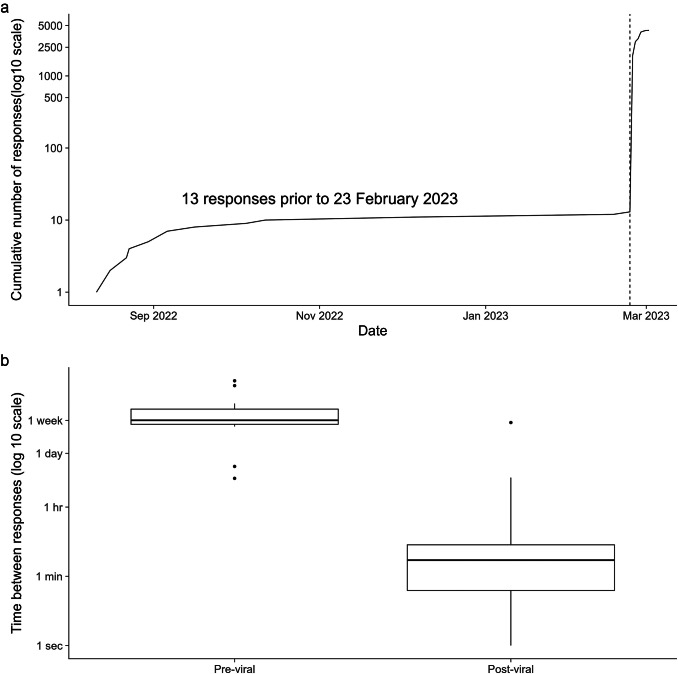
(a) Total number of survey responses recorded between 11 August 2022 and 2 March 2023. (b) The time between survey responses before and after going viral.

In summary, our initial survey to explore app usage among MSM was infiltrated by significant bot activity, rendering the data unusable. Although review of responses by the study team revealed obvious bot activity, some bot responses were more sophisticated and were intermixed with actual human responses – making it difficult to definitively exclude all bot-generated responses. Additionally, while the unusual volume and pattern of responses strongly suggest automated bot activity, it is also possible that some submissions were from real individuals providing intentionally false responses. These may include so-called “survey farm” participants, often based overseas, who complete surveys solely for financial incentives without meeting eligibility criteria [4]. Although our analysis focused on identifying non-human patterns, we acknowledge that both sources may have contributed to the anomalies observed. Regardless, it is essential that online surveys, especially those offering financial incentives as is common when conducting research with populations historically excluded from research efforts, are protected with safeguards that prevent bot infiltration in the first place.

Bot interference is a known phenomenon in online surveys and potential methods to prevent, identify, and filter out these fraudulent responses have been proposed [6]. For example, Griffin et al. disseminated an online Qualtrics survey to explore the impact of the Coronavirus disease 2019 pandemic on the GLBQ+ population that was subsequently infiltrated by bots [7]. The group found that utilizing Qualtrics built-in data safety mechanisms alone, such as CAPTCHA (Completely Automated Public Turing test to tell Computers and Humans Apart), was insufficient and resulted in significant bot activity [7]. They then conducted a second wave of data collection with a variety of alterations to recruitment and survey design, including randomly repeating demographic questions throughout the survey to check for consistency, changing the financial incentive from offering a $5 gift card for each completed survey to raffling ten $100 gift cards, and not using social media to advertise the study – ultimately resulting in the detection of a minimal number of possible bots [7]. Other studies agree that CAPTCHAs alone are insufficient as they can be easily bypassed by bots, especially because programmers can complete the CAPTCHA themselves before activating the bots to complete the remainder of the survey [8]. In addition to CAPTCHA as a first line screen, Storozuk et al. recommends including additional screens, such as having potential participants sign up using a Google form [5]. Participants will only be sent the survey link after they have been pre-screened by the research team [5]. Although CAPTCHA technology as a first line screen is widely recommended, it comes with the trade-off of requiring additional effort for the respondent to enter the survey and can dissuade survey completion due to user frustration [9].

Tran et al. conducted a Qualtrics-based study designed to identify online study-eligible MSM in Philadelphia, Pennsylvania compared using a reference-standard four step approach to a single step geolocation algorithm in detecting bot-generated, fraudulent, duplicate, and geographically ineligible responses [10]. Variations of the four step approach have been used throughout the literature to filter out fraudulent responses [8, 11], with this specific study opting to: 1) remove multiple responses with the same name and/or email address, 2) check for conflicting data between the screening and survey instrument (e.g. discrepancy between self-reported race/ethnicity), 3) remove participants with self-reported ZIP codes outside of Philadelphia, 4) exclude geolocated IP addresses outside of the northeast US [10]. The single step geolocation algorithm only implements step four - mapping IP addresses collected by Qualtrics and excluding participants outside of the designated latitude and longitude [10]. Results showed that geolocation alone provided a moderately high level of agreement with the four-step approach, but could potentially exclude eligible individuals that are traveling when accessing the survey or using a virtual private network [10].

A recent scoping review by Comachio et al. highlights the growing challenge of fraudulent responses in online health research and outlines strategies for identifying and mitigating this issue [12]. Although the review does not specifically address studies focused MSM, we believe that such studies may be particularly susceptible to fraudulent activity given the contexts in which they are conducted. MSM-focused surveys often rely on digital recruitment through dating apps or social media platforms and may include financial incentives - factors that increase visibility to bots or individuals attempting to exploit survey systems. In addition, the sensitive nature of the topics explored may attract opportunistic or malicious responses. Our case offers a real-world example of how quickly fraudulent activity can overwhelm online research, specifically in studies with MSM.

In conclusion, it is clear that a reliable, definitive method for preventing and excluding bot-generated responses without excluding eligible participants is necessary, especially for studies that focus on populations with differing risk and outcomes. In the face of constant advancements in artificial intelligence, researchers must actively protect against bot attacks to ensure rigor and integrity are maintained. Researchers administering surveys with even the potential for online exposure, such as in our case, must implement defensive survey design from the outset [3, 4–8. 11].

## Data Availability

The datasets generated and analyzed during the current study are not publicly available due to concerns regarding participant confidentiality and the inclusion of potentially sensitive demographic and behavioral information. De-identified data may be made available from the corresponding author on reasonable request, contingent upon institutional review and data use agreements.

## Abbreviation

CAPTCHA: Completely Automated Public Turing test to tell Computers and Humans Apart
GLBQ+: gay, lesbian, bisexual, queer, plus
HIV: Human immunodeficiency virus
IP: Internet Protocol
IRB: Institutional Review Board
MSM: Men who have sex with men
REDCap: Research Electronic Data Capture
